# Identification of immune-related biomarkers linked to systemic lupus erythematosus and dilated cardiomyopathy through integrated bioinformatics analysis and multiple machine learning algorithms

**DOI:** 10.3389/fimmu.2025.1606920

**Published:** 2025-07-30

**Authors:** Gaijie Li, Liwen Lin, Shushu Wang, Kachun Lu, KaMan Szeto, Guiting Zhou, Xianwen Tang, Chuanjin Luo

**Affiliations:** ^1^ The First Clinical Medical College, Guangzhou University of Chinese Medicine, Guangzhou, China; ^2^ Department of Cardiology, Shenzhen Hospital of Beijing University of Chinese Medicine (Longgang), Shenzhen, China; ^3^ Cardiology Center, The First Affiliated Hospital of Guangzhou University of Chinese Medicine, Guangzhou, China; ^4^ Cardiovascular Disease, Guangdong Clinical Research Academy of Chinese Medicine, Guangzhou, China

**Keywords:** systemic lupus erythematosus, immune cell infiltration, diagnostic model, bioinformatics analysis, dilated cardiomyopathy

## Abstract

**Background:**

Epidemiological evidence indicates that up to 50% of systemic lupus erythematosus (SLE) patients exhibit cardiac involvement, suggesting a potential strong association between SLE and dilated cardiomyopathy (DCM). This study aims to identify SLE-related genes that may contribute to DCM development and to discover potential biomarkers for early DCM diagnosis in SLE patients.

**Methods:**

We obtained expression profile datasets for dilated cardiomyopathy DCM and SLE from the Gene Expression Omnibus (GEO) database. Through differential expression analysis and weighted gene co-expression network analysis (WGCNA), we screened for candidate biomarkers shared between DCM and SLE and constructed a diagnostic nomogram. The diagnostic performance and effectiveness of the nomogram were evaluated using external datasets and qPCR. Additionally, we performed single-gene set enrichment analysis (GSEA) on key genes to elucidate their potential roles in SLE-related DCM. Finally, we applied the CIBERSORT algorithm to assess immune cell infiltration in both DCM and SLE patients.

**Results:**

Through DEG and WGCNA in the DCM and SLE datasets, we identified a total of 141 key module genes and 24 commonly expressed differentially expressed genes. Enrichment analysis revealed that these 24 genes were primarily involved in inflammation, cell apoptosis, and immune regulation. Through machine learning algorithms and dataset validation, we further identified the HERC6 and IFI44L genes as important diagnostic markers for SLE-related DCM. Experimental validation supports the key role of HERC6, IFI44L, and RSAD2 in SLE-related cardiac dysfunction. Additionally, we developed a nomogram for DCM based on these two genes, and the results showed that both genes exhibited AUC values greater than 0.84. Simultaneously, single-GSEA and immune infiltration analysis indicated immune dysfunction in both DCM and SLE, with both HERC6 and IFI44L significantly associated with immune cell infiltration. Furthermore, connectivity map (cMAP) analysis identified α-linolenic acid as a potential therapeutic agent for treating DCM.

**Conclusion:**

Our study identifies HERC6 and IFI44L as diagnostic markers for DCM in SLE and suggests α-linolenic acid as a potential therapeutic agent.

## Introduction

### Cardiac involvement in systemic lupus erythematosus: clinical manifestations and association with dilated cardiomyopathy

Systemic lupus erythematosus is a multi-system autoimmune disease characterized by the production of autoantibodies, formation of immune complexes, and inflammatory infiltration of multiple organs. Clinically, SLE can affect one or more organs, including the skin, kidneys, joints, nervous system, and heart (1, 2). According to reports, the incidence of cardiac involvement in systemic lupus erythematosus exceeds 50% (3–5). Cardiac involvement in systemic lupus erythematosus can affect the pericardium, myocardium, endocardium, heart valves, and coronary arteries, among others. Pericarditis is the main manifestation of cardiac involvement in SLE (6). Acute lupus myocarditis as the initial symptom is extremely rare in some SLE patients. In most cases, cardiac involvement in systemic lupus erythematosus may not exhibit clinical symptoms, or the symptoms may be mild, atypical, and progress slowly, which poses significant challenges in diagnosing the disease (7). As the disease progresses, long-term myocardial inflammatory infiltration and deposition of immune complexes can lead to structural and functional changes in the heart. According to Jain D, and Halushka MK in their study (8), late-stage SLE-related myocardial disease may present as dilated cardiomyopathy with cardiac chamber enlargement and heart failure (9). Some researchers believe that DCM is one of the most severe organ involvements in SLE (3, 10, 11).

### Pathogenic mechanisms of SLE-related dilated cardiomyopathy: role of immune complexes and inflammatory processes

The mechanisms underlying the combination of SLE and DCM are currently not well understood. In studies where cardiac biopsies were performed on patients with SLE and concurrent cardiac dysfunction, histopathological examination revealed typical manifestations of heart failure such as myocardial cell necrosis and fibrosis. Additionally, immune complex and complement deposition were observed in the perivascular and interstitial areas, along with infiltration of mononuclear cells including lymphocytes, macrophages, and plasma cells (12, 13). It is worth noting that myocardial lesions characterized by myocardial cell necrosis and fibrosis can progress to chronic active myocarditis or even dilated cardiomyopathy (8). In this regard, immune response is believed to be an important pathogenic mechanism in the development of dilated cardiomyopathy in patients with SLE (14). Studies suggest that myocardial damage caused by SLE is primarily an immune complex-mediated vascular phenomenon, leading to complement activation, inflammatory infiltration, and subsequent myocardial injury, rather than direct involvement of the myocardium (7, 10, 15).

### Filling the gap: identifying crosstalk genes and developing diagnostic models for SLE-related DCM

**Figure 1:**
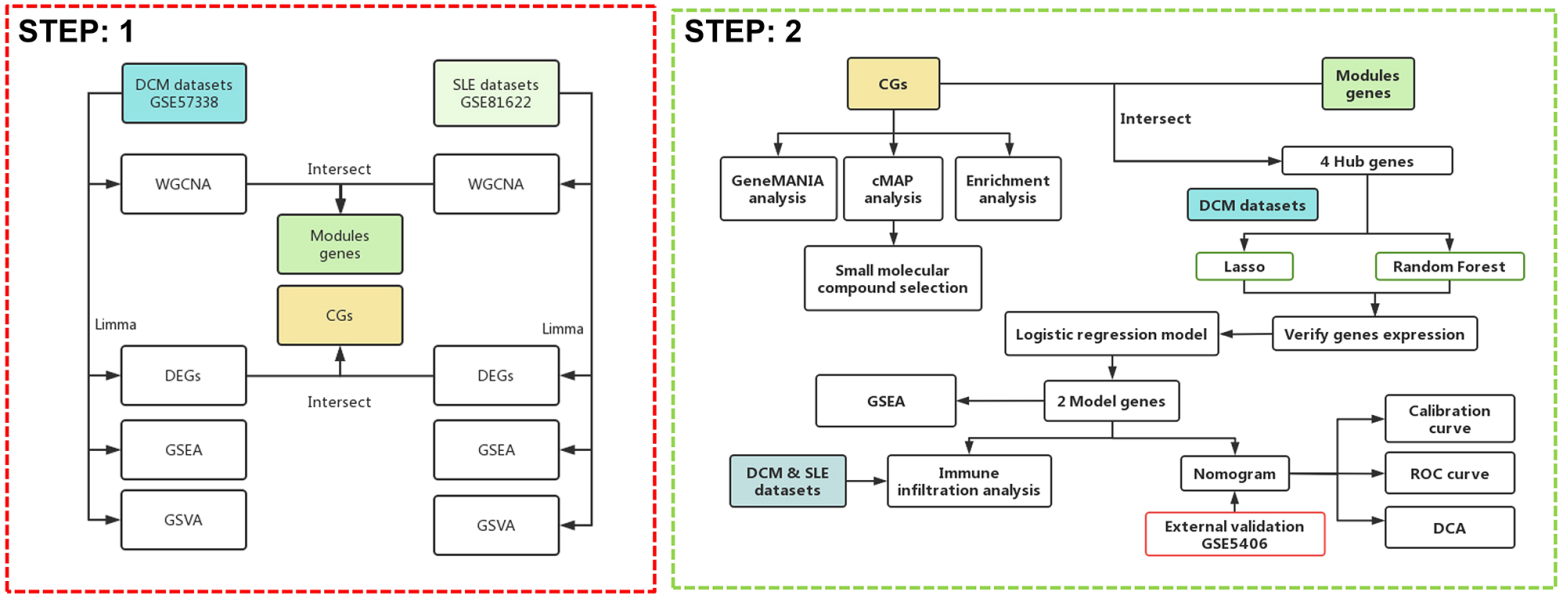
Flowchart of the study design.

Currently, research on SLE complicated with DCM is relatively scarce, and clinical diagnosis remains challenging. There is a need to explore disease mechanisms and identify diagnostic biomarkers to guide diagnosis and treatment, and to prevent the onset and progression of the disease. Therefore, we first utilized RNA-seq and other biological techniques to identify potential crosstalk genes between SLE and DCM, exploring their underlying cellular and molecular mechanisms and assessing their interactions with immune infiltration. This approach enables a deeper understanding of the pathogenic mechanisms of SLE complicated with DCM. Additionally, we employed various machine learning algorithms to further investigate potential diagnostic biomarkers, establish diagnostic models, and evaluate and validate their potential value in disease diagnosis across different cohorts, aiming to fill the existing gaps in the literature. The strategy of bioinformatics analysis is performed as shown in **Figure 1**.

## Methods

### Microarray data collection and processing

The two original expression profile datasets for DCM and control groups, including GSE57338 and GSE5406, were downloaded from the GEO database (https://www.ncbi.nlm.nih.gov/geo/). In the same way, the dataset for SLE and control groups is GSE81622. The GSE57338 dataset (platform: GPL11532) includes 136 control samples (73 male, 63 female) and 82 DCM samples (63 male, 19 female). The GSE5406 dataset (platform: GPL96) includes 16 control samples and 86 DCM samples (the original database did not provide gender information). The GSE81622 dataset (platform: GPL10558) includes 30 SLE samples (4 male, 26 female) and 25 healthy samples (5 male, 20 female). Inclusion Criteria: Datasets must include gene expression data for both SLE or DCM patients. Samples must have been obtained from human subjects. Data must have been generated using high-throughput sequencing technologies. Datasets must include clinical information on the diagnosis of SLE or DCM. Exclusion Criteria: Datasets with incomplete clinical information or missing gene expression data. Studies with a small sample size (less than 15 samples per group).

### Weighted gene co-expression network analysis and identification of key module genes

The “WGCNA” package (16) was used to construct scale-free gene co-expression networks for GSE57338 and GSE81622. The median absolute deviation (MAD) of each gene in both datasets was calculated, and the top 5000 genes with the highest MAD values were selected. The “goodSamplesGenes” function was employed to examine missing entries, low-weighted entries below a threshold, and zero-variance genes in the data. It returned lists of samples and genes with the largest missing values or low-weighted values. In this experiment, the softPower for both datasets was set to 6 as the weighting value. The correlation between the module eigengene matrix and the sample information matrix was calculated. A “labeledHeatmap” was used to visualize the correlation matrix and p-values. The modules with the most significant positive or negative correlation with the module-feature relationship were selected. Finally, the key module genes from both datasets were merged.

### Differential expression gene analysis

DEG analysis was performed using the “Limma” package in R software for the DCM dataset (GSE57338) and SLE dataset. The cut-off criteria for DEGs were set as adj.P.Val < 0.05 and |log(FC)| > 0.5. Subsequently, the expression patterns of DEGs were visualized using the “pheatmap” package and the “ggplot2” package in R software. Heatmaps and volcano plots were generated to represent the expression patterns of DEGs.

### Gene set enrichment and variation analysis

GSEA (17) and Gene set variation analysis (GSVA) (18) can be performed using the R packages “clusterProfiler” and “org.Hs.eg.db”. The “c2.cp.kegg.Hs.symbols.gmt” can be used as a reference gene set. The permutation number can be set as 10,000, and the significance threshold can be set as 10. Finally, the results can be visualized using the R package “enrichplot”.

### GeneMANIA database analysis

To explore the co-expression and interactions of DEGs between SLE and DCM, we utilized the GeneMANIA database (http://genemania.org/). We constructed networks based on co-expression, physical interactions, shared protein domains, and predicted interactions for the common genes (CGs). We employed the “assigned based on query GeneMANIA” strategy to maximize the connectivity among all input genes. Additionally, we used linear regression to automatically select weights that optimize the interaction of genes within the list while minimizing interactions with genes not in the list.

### Function enrichment analysis

To explore the biological functions and specific mechanisms of the disease-related genes in SLE and DCM, we conducted GO and KEGG pathway enrichment analysis on the input gene set (CGs) using packages such as “org.Hs.eg.db”, “GOplot”, “enrichplot”, and “clusterProfiler”. A significance threshold of *p* < 0.05 was used to determine significant enrichment. To visualize the enrichment analysis results, we utilized packages such as “ggplot2”, “circlize”, “RColorBrewer”, and “ComplexHeatmap”. The results of the functional enrichment analysis were presented using circular plots and bubble plots.

### Connectivity map analysis

CMAP (19) (https://clue.io) is a gene expression profile database that is based on gene expression changes induced by perturbations. It has a high predictive value in revealing the relationships between genes and small molecule compounds. In this study, the DEGs shared by DCM and SLE were included in the CMAP online database to identify potential small-molecule drugs for DCM treatment. Subsequently, the top 10 compounds with the highest enrichment scores were identified. The compound descriptions were used to create a Sankey diagram using the “ggalluvial” package.

### Machine learning

To identify potential biomarkers and establish a diagnostic model for DCM, we intersected the DEGs shared by DCM and SLE, as well as the key module genes obtained from the previous WGCNA. Least absolute shrinkage and selection operator (LASSO), a regression approach, facilitates variable selection to heighten the interpretability and predictive precision of a statistical model (20). In our study, LASSO regression was implemented using the “glmnet” package in R, with the optimal regularization parameter (λ) selected through 10-fold cross-validation, aiming to minimize the cross-validated error.

Random Forest (RF) offers advantages such as unconstrained variable conditions and superior accuracy, sensitivity, and specifcity, making it suitable for the prediction of continuous variables and providing consistent forecasts (21). We employed the “randomForest” package in R with 2000 decision trees (ntree = 2000) and default settings for mtry. RF assigns importance scores to variables based on MeanDecreaseGini, which reflects the contribution of each gene to model performance.

To ensure model robustness and mitigate overfitting, both LASSO and RF were conducted independently on the same input gene set, and only genes selected by both methods were retained. Specifically, genes with non-zero coefficients in the optimal LASSO model and MeanDecreaseGini > 6 in the RF model were considered hub genes. These genes were subsequently used to construct a diagnostic model for SLE-associated DCM.

### Validation of the expression levels of key genes in DCM and SLE

The key genes selected based on LASSO regression and RF algorithm analysis were further evaluated for their diagnostic value. A comparison and visualization analysis of the gene expression levels was performed in both the DCM and SLE datasets using the “rstatix,” “ggsignif,” “ggplot2,” and “ggpubr” packages. This analysis aimed to identify genes that were commonly upregulated or downregulated in both DCM and SLE patients.

### Construction of nomograms and evaluation of predictive models for diagnostic markers

Further screening was conducted by performing logistic regression analysis on HERC6, IFI44L, and RSAD2 genes, which exhibited similar expression trends. These genes were identified as common hub genes for both diseases. The “rms” package was used to construct a column line plot. Receiver Operating Characteristic (ROC) curve analysis was performed to evaluate the performance of each hub gene and the column line plot in DCM diagnosis by calculating the area under the curve (AUC). Additionally, ROC curve analysis was conducted to determine if the decision based on the column line plot was beneficial for DCM diagnosis. The efficiency of the column line plot in predicting DCM associated with SLE was evaluated using a calibration curve and decision curve analysis (DCA). Finally, the predictive efficiency of the column line plot was validated in an external DCM dataset, GSE5406.

### Single gene GSEA

GSEA and visualization of the model genes obtained from the aforementioned methods were performed using the “org.Hs.eg.db,” “ggsci,” “patchwork,” and “ggplot2” packages in the GSE57338 dataset. The genes were grouped based on their expression levels, and the significant pathways that influenced the disease were evaluated.

### Immune infiltration analysis

The quantity of immune cell infiltration in the gene expression profiles of DCM and SLE was assessed using the “CIBERSORT” package. The abundance and proportion of immune infiltration for each sample were visualized as bar plots using the “ggplot2” package. The differences in the proportions of 22 immune cell types between DCM and normal samples were compared using the Wilcoxon test, and the results were displayed using a stacked histogram generated by the “ggplot2” package. Furthermore, the associations among the 22 infiltrating immune cell types were visualized using the “corrplot” package, where a p-value < 0.05 was considered statistically significant.

### qPCR validation

Total RNA was extracted from H9C2 cells following the Doxorubicin intervention using a standard TRIzol method. The purity and concentration of the RNA were assessed using a NanoDrop spectrophotometer. cDNA was synthesized from 1 µg of RNA using a reverse transcription kit according to the manufacturer’s protocol. qPCR was performed using SYBR Green Master Mix on an ABI 7500 real-time PCR system. The target genes validated in this study included PIK3IP1, IFI44L, RSAD2, and HERC6, with β-Actin used as an internal control. The primer sequences are shown in **Table 1**.

**Table 1:** Primer sequences.

	Species	Forward	Reverse
HERC6	*Rattus norvegicus*	TGGAGGCAGGAACTGGTCTT	CCTGAATTGGTTCTGGCCGT
IFI44L	*Rattus norvegicus*	AGCATCACCACGCAGTACAA	ATTGGCTCACGTGGGTTGAA
RSAD2	*Rattus norvegicus*	GCCTCCTGATTGAGGGTGAG	CAGTTCAGAAAGCGCATATATTCAT
PIK3IP1	*Rattus norvegicus*	ACACTGGCTGTTCAGTCACC	CCCAGAAGCAGCCTCCAGAT

### Statistical analysis

Continuous variables are expressed as the mean ± SD. Categorical variables are expressed in frequency and percentage (%). Analysis of variance was applied to compare intergroup mRNAs levels. To characterize the diagnostic performance of the mRNAs candidate, ROC curves were applied together with a logistic regression model to determine the AUC and the specificity and sensitivity of the optimal cutoffs. ROC curves were generated by plotting sensitivity against 100-specificity. Data were presented as the AUC and 95%CI. The statistical software package R (www.r-project.org) was used for all analyses.

## Results

### Weighted gene co-expression network analysis and identification of key module genes

**Figure 2:**
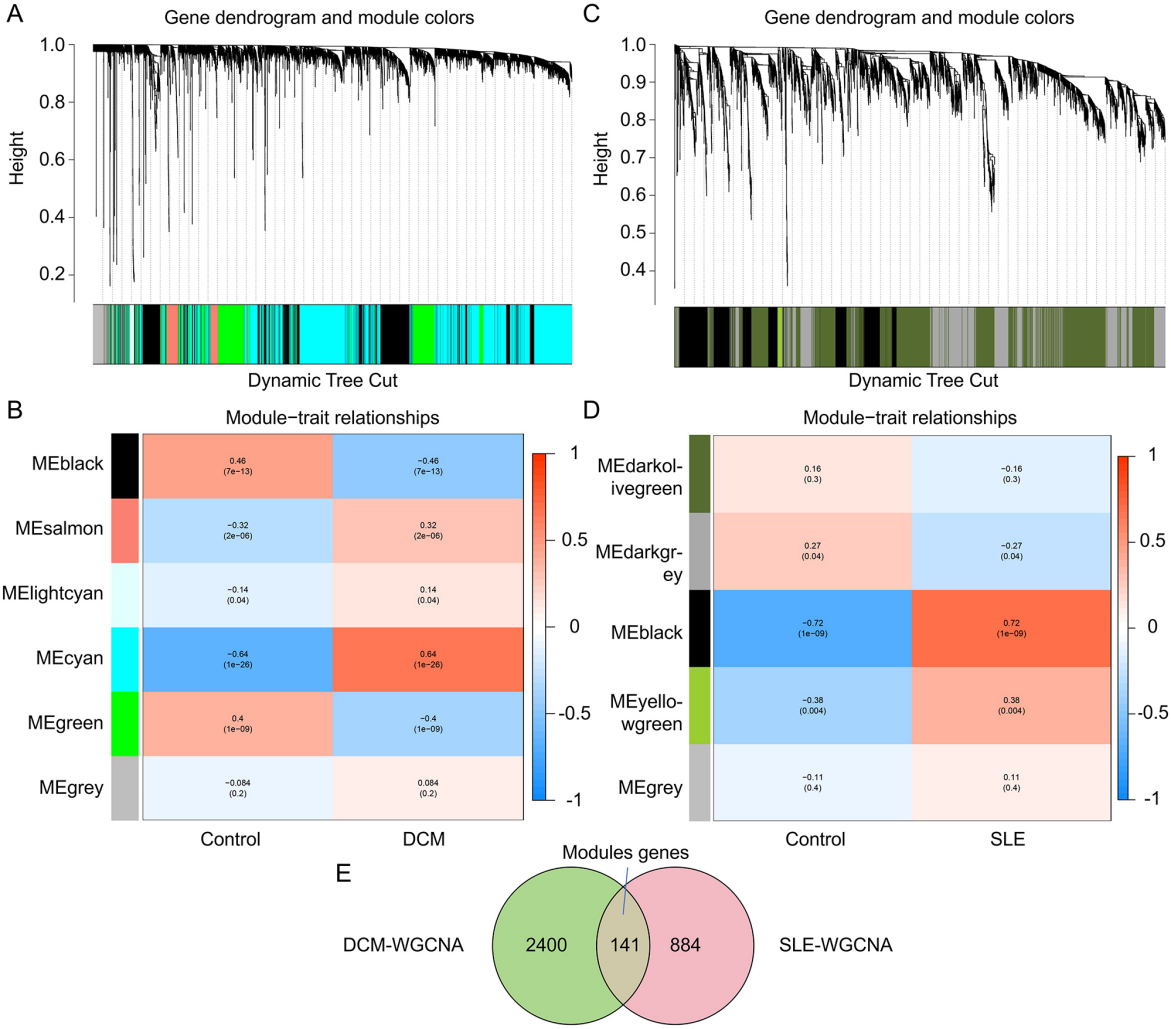
Identification of key module genes in the integrated DCM and SLE datasets using WGCNA. **(A, C)** Hierarchical clustering dendrogram of DCM/SLE gene modules, with different colors representing different modules. **(B, D)** Correlation between module eigengenes and DCM/SLE, where blue indicates a negative correlation and red indicates a positive correlation. **(E)** Venn diagram showing that the cyan module is identified as the key module in the DCM dataset, the black module in the SLE dataset. The intersection of these two modules yields the key module genes termed Modules genes.

To explore the key genes in DCM and SLE, we conducted WGCNA to identify the most relevant gene modules in DCM and SLE samples. In the DCM-WGCNA analysis, a soft-thresholding power (softPower) of 6 was selected based on the scale-free topology criterion. Specifically, softPower = 6 was the lowest power at which the scale-free topology fit index (R²) exceeded 0.85, indicating that the resulting network conformed to a scale-free topology. This resulted in the generation of 6 modules.The cluster dendrogram of the modules is presented in **Figure 2A**. Additionally, we investigated the correlation between DCM and gene modules (**Figure 2B**). The data revealed that the cyan module exhibited the highest positive correlation with DCM (2541 genes, r = 0.64, *p* = 1e−26). Based on this, the cyan module was considered as the key module for subsequent analysis. We also obtained 5 modules through the SLE-WGCNA analysis (**Figures 2C, D**). The black module exhibited the highest positive correlation with SLE (1025 genes, r = 0.72, *p* = 1e−09) so it was considered the key module for further analysis. Additionally, we identified the intersection of module genes, resulting in 141 shared module genes (**Figure 2E**).

### Differentially expressed genes analysis

**Figure 3:**
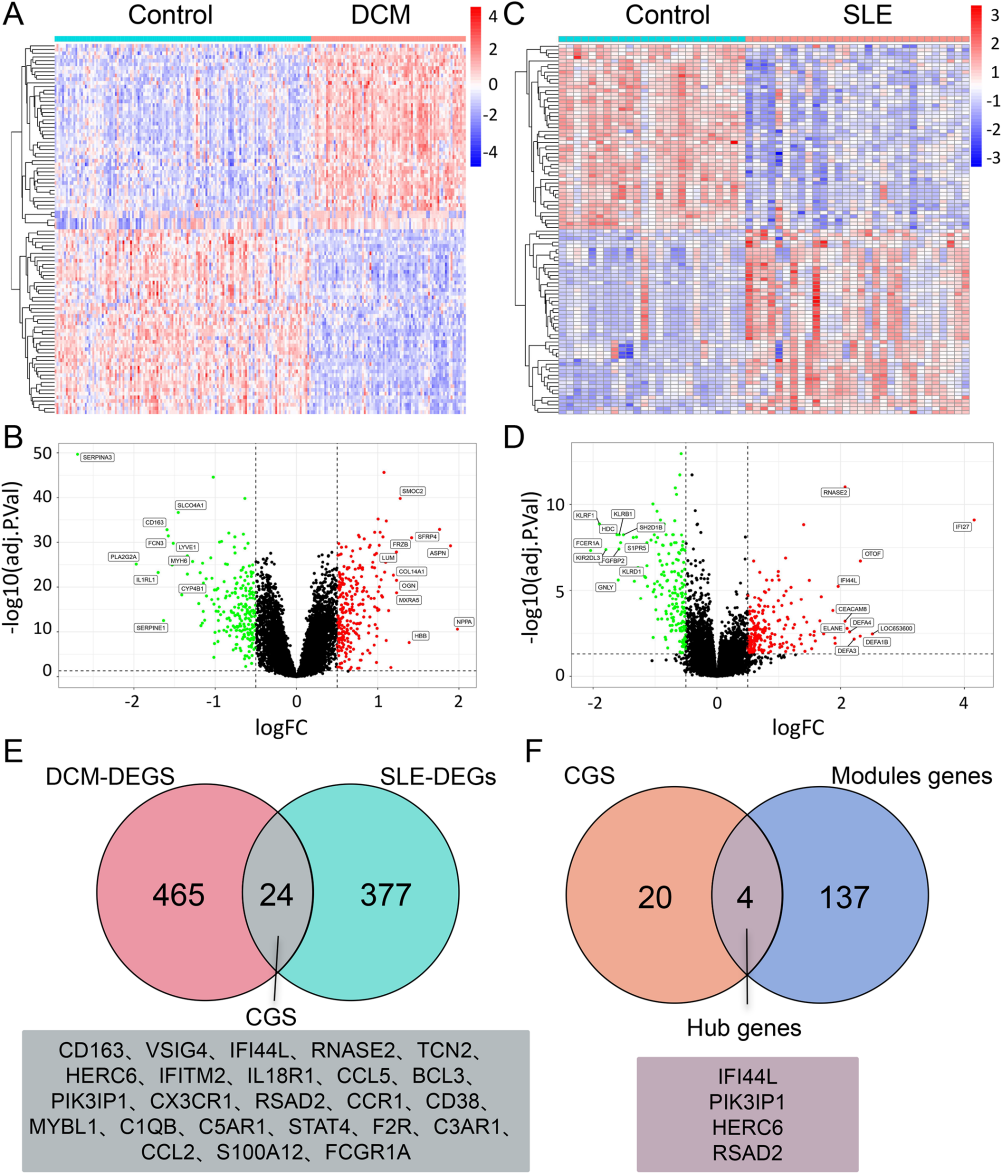
DEG analysis of DCM and SLE datasets. **(A, C)** Heatmap of the top 25 upregulated and 25 downregulated DEGs in the DCM/SLE dataset. **(B, D)** Volcano plot displaying DEGs in the DCM/SLE dataset, with green indicating downregulation and red indicating upregulation. **(E)** Venn diagram intersection of DEGs in DCM and SLE datasets, named CGs. **(F)** Venn diagram intersection of CGs and Module genes, named Hub genes.

DEG analysis was performed using the “Limma” package in R software for the DCM dataset (GSE57338) and SLE dataset. The cut-off criteria for DEGs were set as adj.P.Val < 0.05 and |log(FC)| > 0.5. Differential analysis between DCM and normal samples revealed a total of 489 DEGs, including 269 upregulated genes and 220 downregulated genes. The expression patterns of these DEGs in the DCM dataset were depicted using volcano plots and heatmaps (**Figures 3A, B**). Similarly, there are 401 DEGs between SLE and normal samples, including 213 upregulated genes and 188 downregulated genes (**Figures 3C, D**).To identify the Common genes (CGs) expressed differentially in both DCM and SLE, the VennDiagram package was utilized, revealing 24 CGs (**Figure 3E**). Furthermore, by intersecting the CGs with the Module genes, we identified 4 Hub genes (**Figure 3F**).

### Expression direction of CGs in DCM or SLE datasets

**Figure 4:**
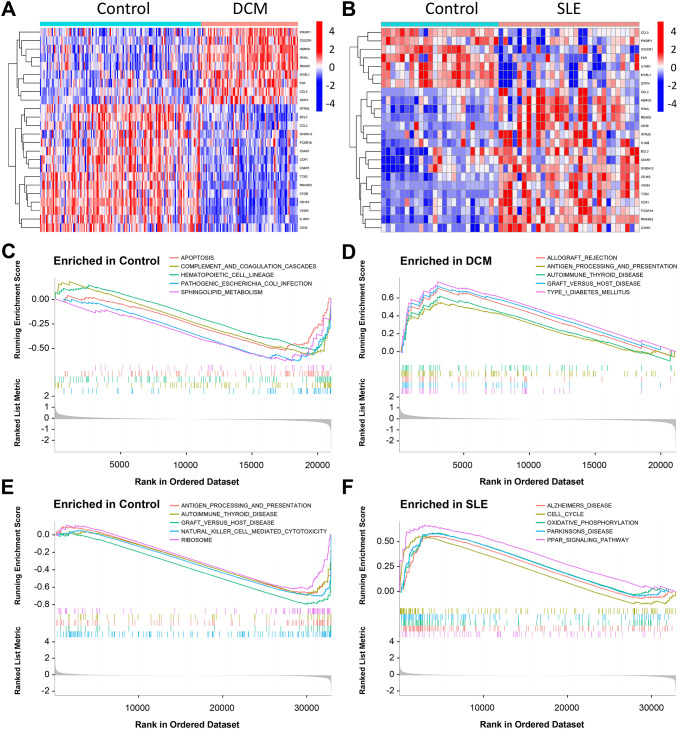
Results of GSEA in the DCM and SLE datasets. **(A, B)** Heatmap displaying the differential analysis of CGs in the DCM dataset (GSE57338) and SLE dataset (GSE81622). **(C, D)** GSEA was performed on the DCM dataset (GSE57338). **(E, F)** GSEA was conducted on the SLE dataset (GSE81622).

The expression patterns of CGs were visualized using the “pheatmap” package in R software. The expression patterns of CGs in DCM patients and normal subjects were shown in **Figure 4A**. The expression pattern of CGs in SLE patients and normal controls is shown in **Figure 4B**.

### Gene set enrichment and variation analysis

The GSEA indicated that the DEGs in the DCM dataset were mainly involved in pathways related to cell apoptosis and immunity, including “Graft Versus Host Disease,” “Complement And Coagulation Cascades,” “lograft Rejection,” “Apoptosis,” “Antigen Processing And Presentation,” and “Sphingolipid Metabolism.” Additionally, enrichment was observed in pathways such as “B Receptor Signaling Pathway” and “Mapkaling Pathway” (**Figures 4C, D**). In the SLE dataset, the DEGs were primarily associated with pathways related to cell cycle and immunity, including “Natural Killer Cell Mediated Cytotoxicity”, “Graft Versus Host Disease”, “Antigen Processing And Presentation,” “T Cell Receptor Signaling Pathway,” “Oxidative Phosphorylation,” and “Cell Cycle” (**Figures 4E, F**). Among them, “Graft Versus Host Disease”, “Antigen Processing And Presentation Autoimmune”, “Thyroid Disease Parkinsons Disease” are pathways shared by both datasets.

The GSVA showed that the DCM dataset was predominantly involved in pathways such as “Vegf Signaling Pathway,” “Sphingolipid Metabolism,” “Apoptosis,” “Graft Versus Host Disease,” “Renin Angiotensin System,” and “Antigen Processing And Presentation” (**Supplementary Figure 1A**). In the SLE dataset, the main pathways included “Glycine Serine And Threonine Metabolism,” “T Cell Receptor Signaling Pathway,” “Antigen Processing And Presentation,” “Graft Versus Host Disease,” “Natural Killer Cell Mediated Cytotoxicity,” “Amino Sugar And Nucleotide Sugar Metabolism,” “Citrate Cycle TCA Cycle,” and “Glycolysis Gluconeogenesis” (**Supplementary Figure 1B**).

### GeneMANIA database analysis

To uncover potential pathogenic genes and mechanisms in SLE-related DCM, we utilized the GeneMANIA database to analyze the interactions among the co-expressed DEGs shared between SLE and DCM. The nodes represent our uploaded genes as well as genes associated with our genes obtained from the GeneMANIA database search. The lines represent different network categories between them. In our network, there are a total of 44 genes, including 24 uploaded genes and 20 related genes, with a total of 1148 connections, including co-expression, physical interaction, shared protein domains, and predicted networks (**Supplementary Figure 1C**).

### Function enrichment analysis

To gain a better understanding of the functions and specific mechanisms of these pathogenic genes, we performed functional enrichment and KEGG pathway analyses on the 14 co-expressed DEGs. Biological Process (BP) analysis of GO revealed that the pathogenic genes in SLE-related DCM are mainly enriched in processes such as “mononuclear cell migration,” “leukocyte chemotaxis,” “T cell differentiation,” and “T cell mediated immunity.” In the Cellular Component (CC) analysis, these genes were mainly located in the “external side of the plasma membrane” and “secretory granule membrane.” In terms of Molecular Function (MF) analysis, the results indicated that “immune receptor activity,” “chemokine receptor binding,” and “complement receptor activity” were the most relevant items among the pathogenic genes (**Supplementary Figure 1D**). KEGG pathway analysis showed that the pathogenic genes in SLE-related DCM are closely associated with pathways such as “Complement and coagulation cascades,” “Viral protein interaction with cytokine and cytokine receptor,” “TNF signaling pathway,” “Cytokine-cytokine receptor interaction,” and “Chemokine signaling pathway” (**Supplementary Figure 1E**).

### Finding candidate small molecule compounds for DCM treatment

**Figure 5:**
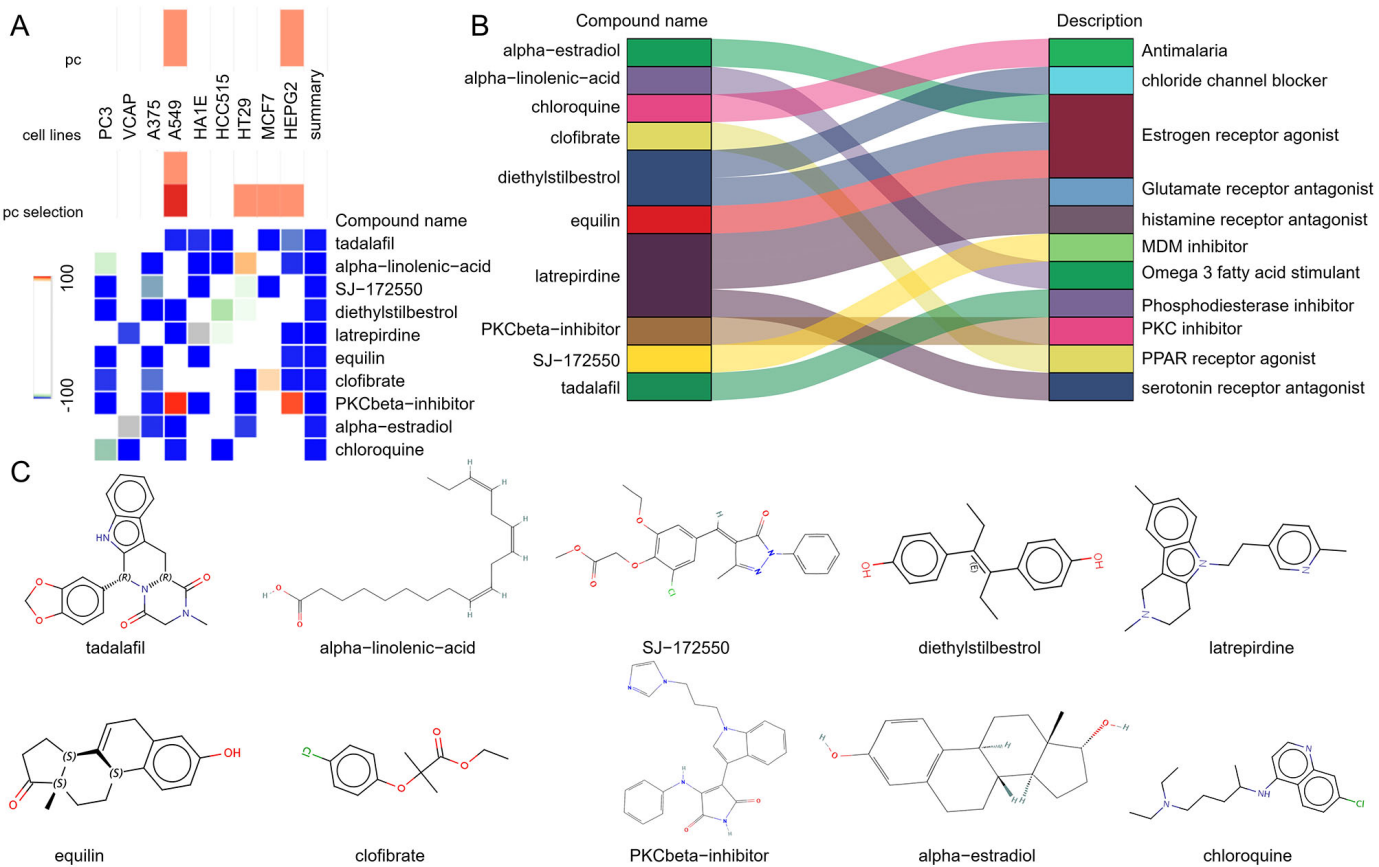
Screening of potential small molecule compounds for the treatment of DCM using CMAP analysis. **(A)** Heatmap displaying the top 10 compounds with the highest enrichment scores based on CMAP analysis in 9 cell lines. **(B)** Description of the top 10 compounds. **(C)** Illustration of the chemical structures of these 10 compounds.

To explore potential small-molecule drugs that may have therapeutic effects on SLE-related DCM patients, we input the DEGs shared between SLE and DCM into the CMAP database to predict small-molecule compounds that can reverse the expression changes of pathogenic genes in DCM. Using the CMAP website (https://clue.io/query) and conducting predictions with CGs, a total of 8559 results were obtained, with 2429 compounds. After excluding drugs without targets, the top ten compounds with the lowest negative scores based on the median tau score were selected. These compounds include tadalafil, alpha-linolenic-acid, SJ-172550, diethylstilbestrol, latrepirdine, equilin, clofibrate, PKCbeta-inhibitor, alpha-estradiol, and chloroquine. They are considered potential therapeutic agents for SLE-related DCM treatment (**Figure 5A**). The targeted pathways and chemical structures of these ten compounds are described in **Figures 5B, C**, respectively.

### Identification of key genes with diagnostic value through machine learning

**Figure 6:**
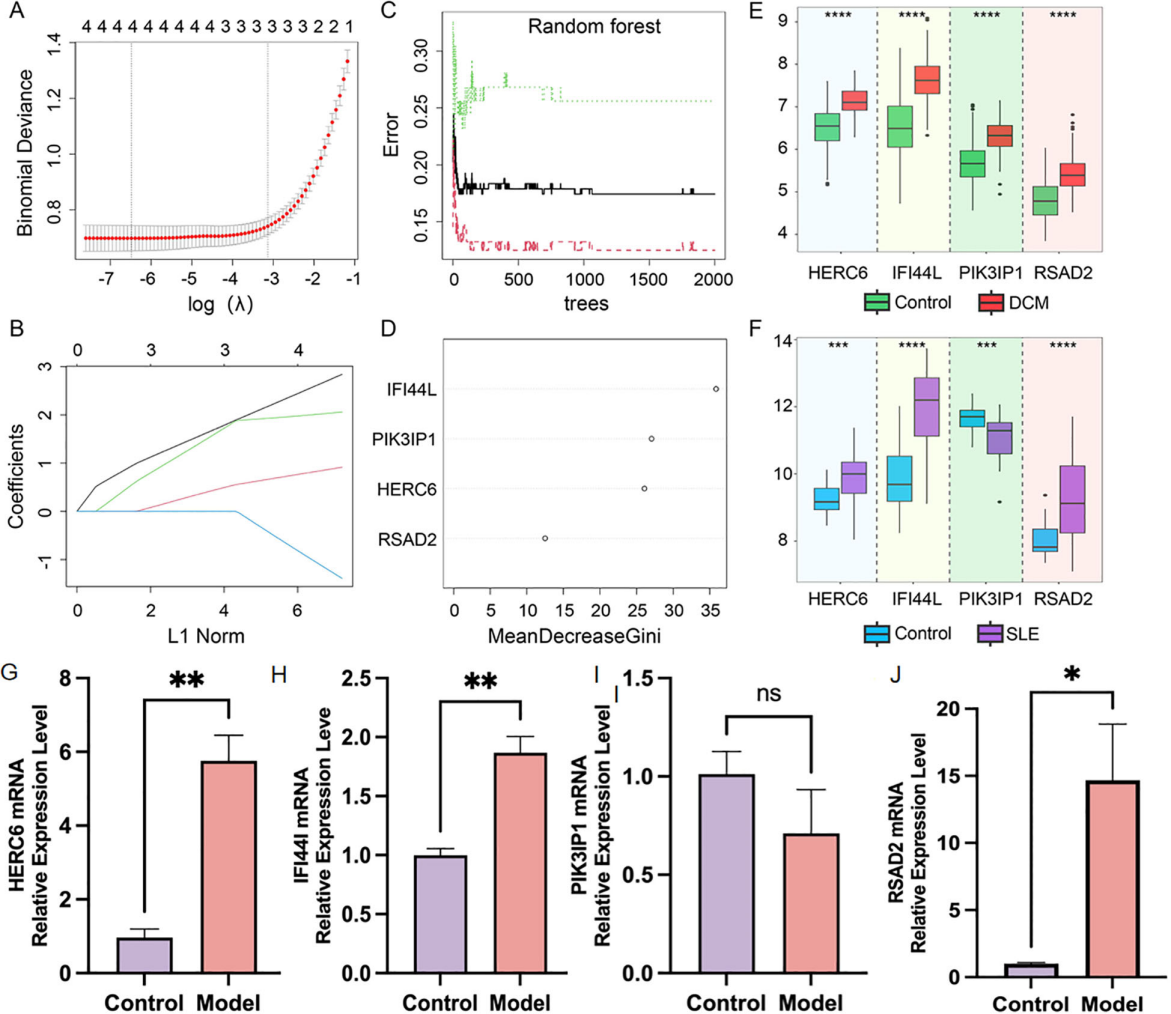
Screening of potential diagnostic biomarkers for SLE-associated DCM using machine learning methods. **(A, B)** Lasso regression analysis was performed on four hub genes to calculate the minimum value **(A)** and λ value **(B)** of diagnostic biomarkers. **(C, D)** The RF algorithm was applied to analyze the four hub genes and a random forest plot was generated. Diagnostic biomarkers were selected based on MeanDecreaseGini scores greater than 6. **(E, F)** Expression profiles of the four hub genes in the DCM dataset GSE57338 and SLE dataset GSE81622. **(G–J)**. Relatively mRNA expression levels of HERC6, IFI44L, PIK3IP1 and RSAD2 in doxorubicin-induced H9C2 cardiomyocytes (*p<0.05, **p<0.01, ***p<0.001, ****p<0.0001).

Due to the potentially crucial role of DEGs shared between DCM and SLE in SLE-related DCM patients, four core genes were identified at the intersection of key module genes in WGCNA for both diseases and the DEGs. The LASSO regression algorithm was applied to these four genes, revealing their significance as potential candidate genes for diagnosing SLE-related DCM patients (**Figures 6A, B**). To further narrow down the scope of diagnostic biomarkers, the RF machine learning algorithm was also employed. The variable importance of each gene was used to rank the four core genes, and genes with MeanDecreaseGini > 6 were selected (**Figures 6C, D**). Interestingly, these four genes remained influential in diagnosing SLE-related DCM according to the RF model.

### Validation of the expression levels of key genes in DCM and SLE

As shown in **Figure 6E**, in the DCM dataset GSE57338, the expression levels of HERC6, IFI44L, PIK3IP1, and RSAD2 were significantly higher in DCM patients compared to the control group (*P*<0.05). In **Figure 6F**, in the SLE dataset GSE81622, the expression levels of HERC6, IFI44L, and RSAD2 were significantly higher in SLE patients compared to the control group (*P*<0.05); whereas the gene expression level of PIK3IP1 was significantly lower in SLE patients compared to the control group (*P*<0.05). Therefore, the expression levels of HERC6, IFI44L, and RSAD2 were upregulated in both DCM and SLE patients.

Furthermore, we validated the expression of the aforementioned genes in doxorubicin-induced H9C2 cardiomyocytes. Compared to the control group, the model group showed significantly elevated expression levels of HERC6, IFI44L, and RSAD2 (P<0.05), with HERC6 being the most pronounced, while PIK3IP1 expression exhibited no significant difference (**Figures 6G–J**).

### Construction of the nomogram and evaluation of the diagnostic biomarker prediction model

**Figure 7:**
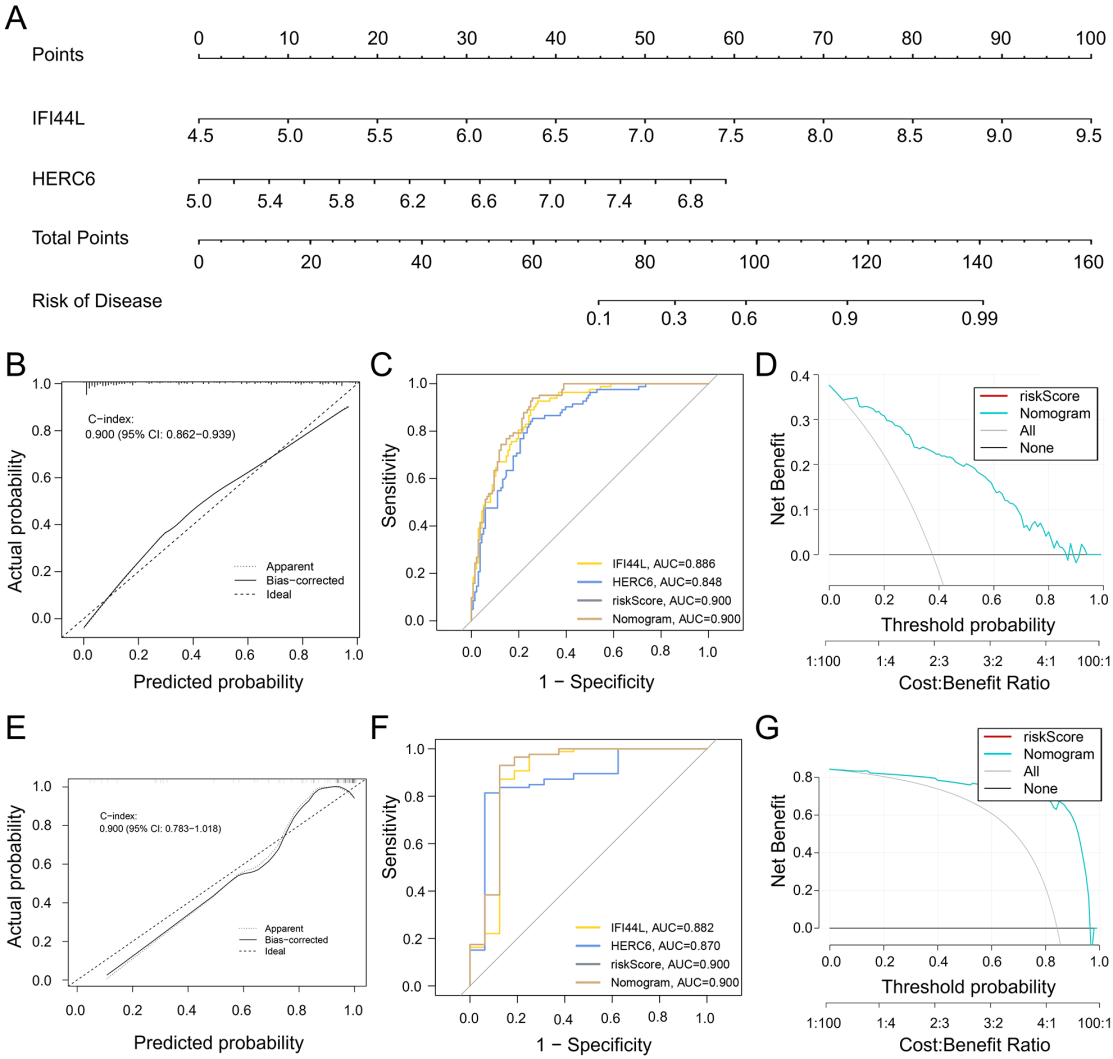
Development and efficacy evaluation of a diagnostic line graph model. **(A)** After performing logistic regression on three genes (HERC6, IFI44L, and RSAD2), further screening identified HERC6 and IFI44L as the two key genes for constructing the diagnostic line graph. **(B)** Calibration curves of the column line graph model predicted in SLE-associated DCM. The dashed line labeled “Ideal” represents the standard curve. The dotted line labeled “Apparent” represents the uncalibrated prediction curve, while the solid line labeled “Bias-corrected” represents the calibrated prediction curve. **(C)** ROC of the diagnostic performance of the two candidate biomarkers (HERC6 and IFI44L). **(D)** DCA for the nomogram model. The black line is marked as “None”, which stands for the net benefit of the assumption that no patients have DCM. The grey line is marked as “All”, which indicates the net benefit of the assumption that all patients have DCM, and the purple line is marked as “Nomogram”, and represents the net benefit of the assumption that SLE-related DCM is identified according to the diagnostic value of DCM predicted by the nomogram model. **(E–G)** Validation of the calibration curve, ROC curve, and decision curve analysis (DCA) of the column line graph in the external validation set GSE5406 for DCM.

To better facilitate diagnosis and prediction, we conducted logistic regression analysis on HERC6, IFI44L, and RSAD2, which exhibited similar expression trends. After further screening, we identified two key core genes, namely HERC6 and IFI44L. Based on this, we constructed a Nomogram (**Figure 7A**). The calibration curve of the constructed nomogram diagnostic model showed that the predicted probabilities were nearly identical to those of the ideal model, with a C-index of 0.900 (95% CI: 0.862-0.939) (**Figure 7B**). We evaluated the AUC values of each core gene and the nomogram using ROC to determine their sensitivity and specificity in diagnosing SLE-related DCM. As expected, the AUC values of both core genes were greater than 0.84, while the AUC value of the nomogram was higher than that of each individual core gene, suggesting that the nomogram may have a strong diagnostic value for SLE-related DCM (**Figure 7C**). Additionally, decision curve analysis (DCA) was performed to assess the clinical utility of the nomogram. The results showed that decision-making based on the nomogram model may contribute to the diagnosis of SLE-related DCM (**Figure 7D**).

To validate the nomogram, we used the GSE5406 dataset from the GEO database, which also included DCM patients, as an external validation set. The ROC curve, DCA, and calibration curve of the nomogram all indicated good diagnostic performance for SLE patients with DCM (**Figures 7E–G**).

### Single gene GSEA

**Figure 8:**
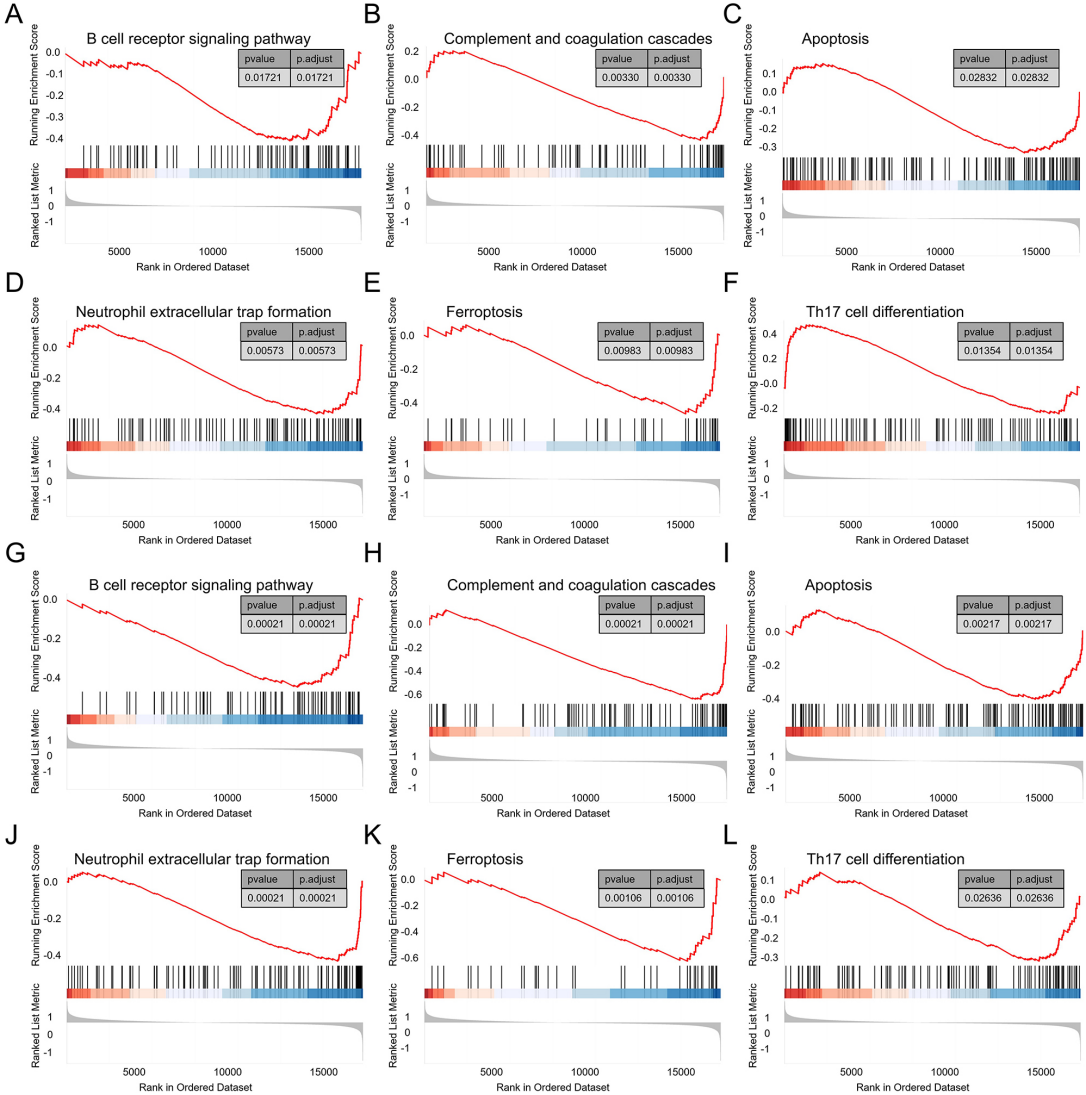
Single gene GSEA. **(A–F)** GSEA results of IFI44L in the DCM dataset GSE57338. **(G–L)** GSEA results of HERC6 in the DCM dataset GSE57338.

In the GSEA analysis on the DCM dataset, 81 pathways, including “B cell receptor signaling pathway,” “Complement and coagulation cascades,” “Apoptosis,” “Neutrophil extracellular trap formation,” “Ferroptosis,” “Th17 cell differentiation,” and “Cellular senescence,” were identified as regulatory targets of IFI44L (**Figures 8A–F**). In the single-gene GSEA analysis of MID1IP1, 114 pathways were determined as regulatory targets of HERC6, which also included “B cell receptor signaling pathway,” “Complement and coagulation cascades,” “Apoptosis,” “Neutrophil extracellular trap formation,” “Ferroptosis,” “Th17 cell differentiation,” and “Cellular senescence” (**Figures 8G–L**).

### Infiltration of immune cells in DCM and its correlation with invasive immune cells analysis

**Figure 9:**
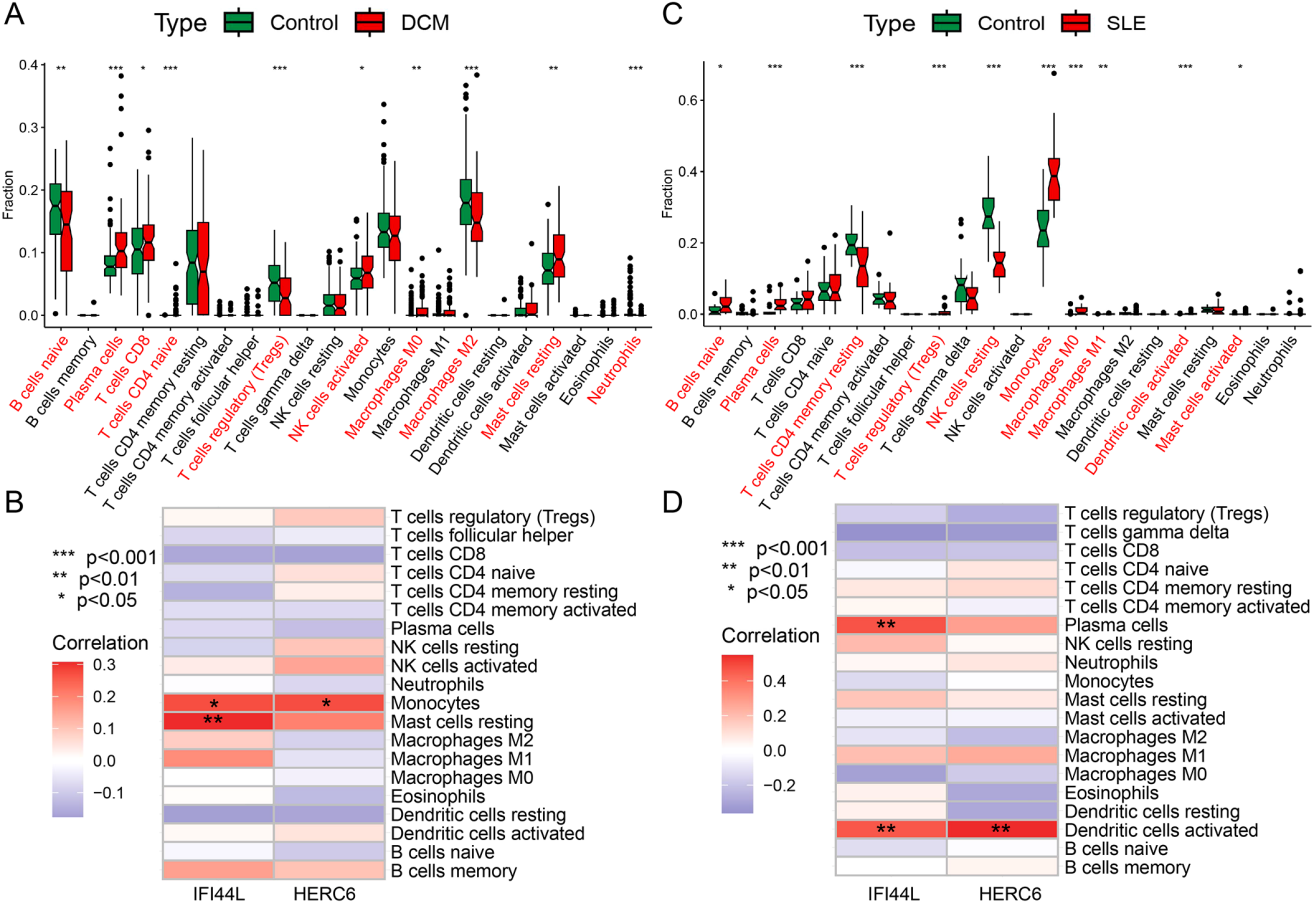
Immune cell infiltration analysis in DCM and SLE **(A, C)** The box plot displays a comparison of 22 immune cell types between the DCM/SLE group and the control group. **(B, D)** At a threshold of *p* < 0.05, it indicates the association between differentially infiltrating immune cells in DCM/SLE and two hub genes. (*p < 0.05, **p < 0.01, ***p < 0.001).

Functional and pathway analysis of DCM and SLE-related pathogenic genes revealed a close association with inflammation and immune processes. To investigate the result, we utilized the CIBERSORT algorithm to infer the characteristics of immune cells and explore the correlation between immune cell infiltration and immune regulation as well as diagnostic biomarkers in DCM and SLE. **Figure 9A** exhibits the proportions of 22 immune cell types in DCM and normal samples from the GSE57338 dataset, showing significant differences in 10 immune cell subtypes. Compared to the control group, DCM exhibited higher proportions of Plasma cells, T cells CD8, T cells CD4 naive, NK cells activated, Macrophages M0, and Mast cells resting, while lower proportions of B cells naive, T cells regulatory (Tregs), Macrophages M2, and Neutrophils. Similarly, we found significant differences between SLE and normal samples across 10 immune cell subtypes (**Figure 9C**). Compared to the control group, SLE exhibited higher proportions of B cells naive, Plasma cells, T cells regulatory (Tregs), Monocytes, Macrophages M0, Macrophages M1, Dendritic cells activated, and Mast cells activated, while lower proportions of T cells CD4 memory resting and NK cells resting. Finally, we discovered that both key genes, IFI44L and HERC6, were significantly correlated with immune cell accumulation in both DCM and SLE (**Figures 9B, D**).

## Discussion

Cardiac involvement is one of the three major causes of mortality in SLE (22). SLE can invade the myocardium, leading to long-term inflammatory infiltration and deposition of immune complexes, which can result in dilated cardiomyopathy (23). However, early myocardial damage is often insidious and difficult to identify based on clinical symptoms alone, despite the use of endomyocardial biopsy, which still has low sensitivity (24, 25). When patients seek medical attention due to the appearance of symptoms, myocardial damage has often already progressed to dilated cardiomyopathy, subsequently leading to heart failure. Clinical studies have conducted follow-ups on patients with lupus myocarditis and reported a mortality rate of 10% after a 3-year follow-up period (26). Early identification and diagnosis of SLE with concurrent dilated cardiomyopathy are crucial steps in halting disease progression and improving prognosis. Our study utilized various bioinformatics techniques to uncover the potential pathogenic mechanisms of inflammation and immune response in SLE-associated DCM. It also developed a diagnostic nomogram based on IFI44L/HERC6 and identified alpha-linolenic acid as a potential therapeutic drug for SLE-associated DCM.

In this study, we conducted a comprehensive analysis of transcriptional data from cardiac tissue of patients with DCM and peripheral blood mononuclear cells (PBMCs) from patients with SLE. DCM is considered an organic lesion, thus tissue samples offer greater accuracy in predicting transcriptional variations associated with lesions compared to circulating samples. While SLE is marked by abnormal immune activation and circulating organ damage. Blood PBMCs, comprising diverse immune cells, provide crucial transcriptional insights into SLE’s effects on target organs. Using the expression matrix of SLE and DCM genes, we identified a total of 24 potential interacting genes (CG). Functional analysis revealed that these CGs were primarily enriched in immune and inflammatory-related pathways, closely associated with immune cell migration and chemotaxis, immune complement and receptor activity, as well as inflammatory infiltration. Previous studies suggest that autoimmune reactions and inflammation contribute to myocarditis and its progression to dilated cardiomyopathy, with persistent autoimmune activity underlying its development (27). SLE, as a systemic autoimmune disease, is characterized by tissue-specific alterations that lead to T-cell-dependent production of anti-nuclear antibodies. These alterations are accompanied by immune complex deposition, expression of chemotactic factors, and activation of T lymphocytes, resulting in the production of cytokines (28). In SLE-induced myocardial damage, T cells, particularly CD4+ T cells, play a key role by releasing inflammatory mediators like TNF-α and IFN-γ, causing cardiac inflammation and injury (29). This study found CD4+, CD8+ T cells, and activated macrophages in the hearts of dilated cardiomyopathy (DCM) patients, consistent with previous findings. Dysregulated B cells increase autoantibody and cytokine production, activating complement and forming immune complexes that damage cardiac tissue (30). Reduced CR1 in SLE impairs immune complex clearance, leading to sustained inflammation (31). The dysregulation of immune complex dissolution and clearance further exacerbates the accumulation of immune complexes in the myocardium, leading to sustained myocardial inflammation. Through signaling from chemokine receptors, monocyte macrophages can be guided to myocardial tissue, participating in the regulation of inflammatory responses and cellular engulfment, clearing damaged cells and immune complexes. In a spontaneous SLE mouse model, infiltration of monocytes in the myocardium and focal necrosis of muscle fibers can be observed (32). In SLE cardiac pathology, lymphocytes, macrophages, plasma cells, and immune complexes (IgG, C1q) infiltrate the myocardium, intensifying inflammation and worsening cardiac function. This process can eventually lead to dilated cardiomyopathy and heart failure (33).

Using CIBERSORT, we further analyzed the immunological patterns of SLE and DCM. The results revealed a higher proportion of Plasma cells and macrophage M0 infiltration in both DCM and SLE samples. Additionally, there was a disruption in the balance between macrophage subtypes in both SLE and DCM. In SLE, there was a significant increase in the infiltration of pro-inflammatory M1 macrophages, while in DCM samples, there was a notable decrease in the infiltration of anti-inflammatory M2 macrophages. Furthermore, the results indicated a decrease in the proportion of regulatory T cells (Tregs) in DCM samples. Interestingly, an elevated proportion of Tregs was observed in SLE samples. Tregs are crucial for suppressing pathological immune responses and maintaining immune homeostasis (34). A low number of Tregs can lead to immune hyperactivation (35), which aligns with the reduced infiltration of Tregs in DCM. However, the increase in SLE Tregs is puzzling, as studies show inconsistent Treg levels in SLE patients across different populations. These discrepancies may stem from the lack of specific Treg markers and the functional complexity of various Treg subtypes (36).

To ensure the robustness of our results, we applied We apply a variety of machine learning algorithms to screen for core genes. The results identified HERC6 and IFI44L as key genes for developing a diagnostic model. HERC6, an E3 ubiquitin ligase, is crucial in immune regulation and antiviral defense, with its abnormal expression linked to immune hyperactivation in SLE with DCM (37) ^(^ 38 ^),^. IFI44L, an interferon-regulated gene, plays a role in regulating viral replication and dissemination (39). In addition to its antiviral role, IFI44L is also associated with biological processes such as cell apoptosis and immune regulation (40, 41). Both genes correlate with immune cell accumulation in SLE. Notably, HERC6 and IFI44L may synergistically contribute to immune dysregulation in SLE-DCM. HERC6 can enhance the degradation or modification of key immune signaling molecules via ubiquitination, thereby influencing the interferon pathway. IFI44L, as an interferon-stimulated gene, amplifies type I interferon signaling and downstream immune responses. Their concurrent upregulation may form a positive feedback loop, promoting sustained immune activation, excessive cytokine release, and tissue inflammation in cardiac tissue. This interaction potentially drives the chronic inflammatory environment observed in SLE-associated cardiomyopathy.

Current diagnostic paradigms for SLE-related cardiac involvement primarily depend on imaging techniques including echocardiography and cardiac MRI, supplemented by nonspecific biomarkers such as troponin and NT-proBNP (42, 43). These conventional approaches exhibit notable constraints in early disease detection. Echocardiographic abnormalities typically manifest only after considerable myocardial damage has already ensued. Although cardiac MRI demonstrates greater sensitivity, its high cost precludes widespread use as a screening tool. While serological markers offer clinical accessibility, their limited disease specificity and weak correlation with subclinical myocardial injury reduce diagnostic reliability.

Our nomogram demonstrated strong diagnostic performance for SLE-induced DCM, confirmed through validation with external datasets. Moreover, experimental validation supported the involvement of HERC6, IFI44L, and RSAD2 in SLE-related cardiac dysfunction, with HERC6 showing the most pronounced upregulation in doxorubicin-treated H9C2 cardiomyocytes. In contrast, the expression of PI3K3IP1 remained unchanged, suggesting its role may be limited or context-dependent in this pathological process.

This study also screened potential small molecule drugs with therapeutic effects on SLE-associated DCM. The results showed that tadalafil, alpha-linolenic acid, SJ-172550, diethylstilbestrol, latrepirdine, equilin, clofibrate, PKCbeta-inhibitor, alpha-estradiol, and chloroquine are potential therapeutic drugs. Among them, alpha-linolenic acid exhibited a significant negative score in cMAP analysis, indicating its potential efficacy in reversing the expression changes of SLE-associated pathogenic genes in DCM. Alpha-linolenic acid is an omega-3 polyunsaturated fatty acid (PUFA) belonging to the linolenic acid family. It can be converted into other longer-chain omega-3 fatty acids in the human body. Numerous studies have shown that alpha-linolenic acid, as a precursor of omega-3 fatty acids, can reduce the risk of cardiovascular diseases and exert various anti-inflammatory and antiplatelet aggregation effects in conditions like atherosclerosis, viral myocarditis, and systemic lupus erythematosus (44–46). Mechanistically, alpha-linolenic acid has been reported to inhibit the NF-κB signaling pathway and reduce the expression of pro-inflammatory cytokines such as IL-6 and TNF-α (47). It may also modulate the activity of peroxisome proliferator-activated receptors (PPARs), which play a critical role in suppressing inflammation and improving endothelial function (48). These pathways are highly relevant to the inflammatory and immune dysregulation observed in SLE-associated cardiac injury. Therefore, as a precursor of omega-3 fatty acids, alpha-linolenic acid may exert a positive impact on the treatment of SLE-associated DCM by regulating cardiac inflammation responses and cellular signaling pathways. However, further experimental validation and clinical research are needed to elucidate its specific mechanisms and effectiveness in the treatment of SLE-associated DCM.

Despite the valuable findings of this study, there are some limitations to consider: (1) Data sources and sample size: This study primarily relied on RNA-seq data from public databases, which may affect data quality and reliability. The relatively small sample size and the significant gender imbalance in the GSE57338 dataset may introduce sex-related bias in gene expression analysis and limit the generalizability of the findings. Moreover, although we applied standard normalization and batch effect correction methods, residual batch effects from different datasets may still confound the results. (2) Validation of biomarkers: HERC6 and IFI44L were selected as candidate biomarkers in this study, but their validation and application in clinical practice require further support from clinical experiments. (3) Limitations of cMAP analysis: While CMAP analysis provides clues for potential therapeutic drugs, its results are solely based on existing datasets and require further validation in laboratory and clinical settings. (4) Insufficient mechanistic insights: This study did not delve into the mechanisms underlying SLE complicated with DCM. And The use of transcriptional data from different tissue sources, cardiac tissue for DCM and PBMCs for SLE, may introduce bias and limit the direct comparability of the results. Further research is needed to explore the cellular and molecular mechanisms in the future. (5) Model limitations: Functional validation was conducted using H9C2 cells, a rat cardiomyoblast cell line that does not fully replicate the physiological characteristics of adult human cardiomyocytes (49). Therefore, findings from this model should be interpreted with caution and further verified in more clinically relevant human-derived systems.

In future research, efforts should focus on increasing sample sizes, performing systematic experimental validations, and further investigating the molecular mechanisms underlying SLE-associated DCM. In addition, the clinical applicability of the proposed diagnostic model based on immune-related gene expression profiles should be evaluated in prospective studies. Compared with conventional diagnostic approaches such as imaging techniques and nonspecific serological markers, which often lack sensitivity in early-stage disease, this transcriptomic model may provide a complementary tool for the early detection of SLE-related cardiac involvement. Potential clinical applications include the development of a blood-based PCR assay or incorporation of the model into artificial intelligence–driven risk assessment systems. However, these strategies require further validation across diverse clinical cohorts to ensure their feasibility and reliability.

## Data Availability

Publicly available datasets were analyzed in this study. This data can be found here: The data presented in this study are openly available in Gene Expression Omnibus (GEO) (URL: https://www.ncbi.nlm.nih.gov/geo/, accessed on 13 November 2023).

## Supplementary material

Supplementary Figure 1
**(A)** Venn diagram of GSEA results for DCM and SLE datasets. **(B)** GSVA were performed on the DCM dataset (GSE57338). **(C)** GSVA were performed on the SLE dataset (GSE81622). **(D)** GeneMANIA database analysis resulted in a network consisting of 44 genes and 1148 connections, including Co-expression, Physical Interaction, Shared protein domains, and Predicted networks. **(E)** The circular plot illustrated the GO enrichment analysis. **(F)** The chord diagram represented the KEGG enrichment analysis.
