# Supplementary figures and images for: Identification of immune-related biomarkers linked to systemic lupus erythematosus and dilated cardiomyopathy through integrated bioinformatics analysis and multiple machine learning algorithms
Source: Front Immunol. 2025 Jul 30;16:1606920. doi: 10.3389/fimmu.2025.1606920 (PMC12343684; doi:10.3389/fimmu.2025.1606920)

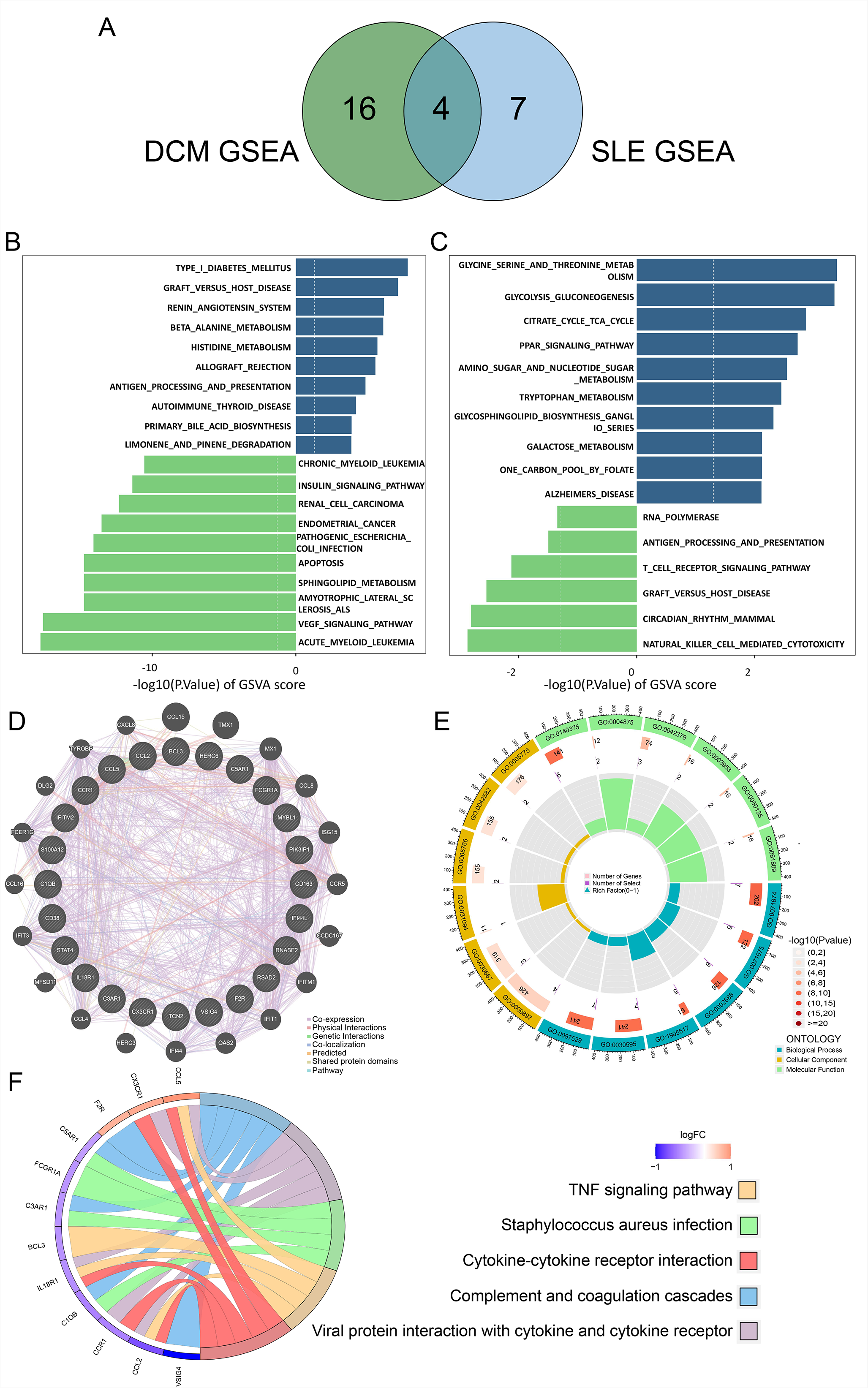

Supplement: Supplementary Figure 1 — (A) Venn diagram of GSEA results for DCM and SLE datasets. (B) GSVA were performed on the DCM dataset (GSE57338). (C) GSVA were performed on the SLE dataset (GSE81622). (D) GeneMANIA database analysis resulted in a network consisting of 44 genes and 1148 connections, including Co-expression, Physical Interaction, Shared protein domains, and Predicted networks. (E) The circular plot illustrated the GO enrichment analysis. (F) The chord diagram represented the KEGG enrichment analysis. [file Image1.tif]
